# Clinical characteristics, gestational weight gain and pregnancy outcomes in women with a history of gestational diabetes mellitus

**DOI:** 10.1186/s13098-021-00694-9

**Published:** 2021-07-06

**Authors:** Xin Liang, Wei Zheng, Cheng Liu, Lirui Zhang, Li Zhang, Zhihong Tian, Guanghui Li

**Affiliations:** Division of Endocrinology and Metabolism, Department of Obstetrics, Beijing Obstetrics and Gynecology Hospital, Capital Medical University, No 251, Yaojiayuan Road, Chaoyang District, Beijing, 100026 China

**Keywords:** A history of gestational diabetes mellitus, Glucose and lipid profiles, Gestational weight gain, Pregnancy outcomes

## Abstract

**Background:**

Pregnant women with a history of gestational diabetes mellitus (GDM) are at high risk of GDM. It is unclear whether this population has pregnancy characteristics different from the general population. Whether these features affect the perinatal outcome has not yet been elucidated.

**Methods:**

A retrospective study was conducted, including baseline characteristics, laboratory data, gestational weight gain (GWG), and pregnancy outcomes of 441 pregnant women with prior GDM. Besides, 1637 women without a history of GDM treated in the same period were randomly selected as the control group. The above indicators of the two groups were compared. Multivariable logistic regression analysis was performed to investigate how GWG was associated with perinatal outcomes for previous GDM women.

**Results:**

Among women with GDM history, triglycerides (TG) and fasting plasma glucose (FPG) in the 1st trimester were higher than those without GDM history. GWG was lower in women with prior GDM relative to the control group at various pregnancy stages. However, women with GDM history had a higher risk of developing GDM (OR 3.25, 95% CI 2.26–4.68) and pregnancy-induced hypertension (OR 1.50, 95% CI 1.05–2.45). In women with previous GDM, excessive GWG before OGTT exhibited a positive correlation with pregnancy-induced hypertension (OR 1.47, 95% CI 1.05–3.32), while inadequate GWG was not a protective factor for GDM and pregnancy-induced hypertension.

**Conclusion:**

Women with prior GDM have glucose and lipid metabolism disorders in the 1st trimester. Limited reduction of GWG before oral glucose tolerance test (OGTT) was insufficient to offset the adverse effects of glucose and lipid metabolism disorders in women with previous GDM. Relevant interventions may be required at early stage or even before pregnancy.

## Background

Gestational diabetes mellitus (GDM) is a prevalent pregnancy metabolic disorder characterized by insulin resistance and impaired pancreatic β-cell function [1, 2]. It can lead to adverse perinatal outcomes and influence long-term health of mothers and their offspring [3]. In recent years, the incidence of GDM in CHINA has reached 17.5–18.9% [4]. The promotion of “two-child” policy in China has recently significantly increased the rates of high-risk pregnancy and adverse pregnancy outcomes [5, 6]. Particularly, the number of pregnant women with GDM history has increased, and previous studies suggest that GDM history is a risk factor for the recurrence of GDM and type 2 diabetes [7, 8]. It was reported that women with prior GDM had a tenfold increased risk for type 2 diabetes compared to those without GDM history [9]. There was a higher likelihood of developing type 2 diabetes in women who were overweight or obese before pregnancy with a history of GDM compared to their counterparts without a history of GDM [10]. Women with GDM history are at high risk for recurrence of GDM and the development of type 2 diabetes, however, the general condition of their subsequent pregnancy remains unclear. Most articles have focused on postpartum metabolic characteristics of women with GDM history, but less attention has been given to the differences between women with previous GDM and the general pregnant women during a subsequent pregnancy. In this regard, our study attempted to elucidate different clinical characteristics, glucose metabolism, lipid metabolism, gestational weight gain (GWG) at various stages of pregnancy, and pregnancy outcomes of women with GDM history and those without GDM history. Meanwhile, we investigated GWG effect before oral glucose tolerance test (OGTT) on pregnancy outcomes of women with prior GDM, aiming to produce relevant information to guide proper management methods during pregnancy and reduce related adverse maternal and infant outcomes.

## Materials and methods

### Study subjects

We conducted a retrospective cohort study comprising pregnant women who established a medical record for receiving healthcare and delivered singleton gestations at Beijing Obstetrics and Gynecology Hospital affiliated to Capital Medical University from January 1, 2017 until December 31, 2018. On the one hand, participants were included in the study if they; (i) were multipara; (ii) were women who used to diagnosed with GDM after 2011. On the other hand, subjects were excluded if they; (i) were aged > 45 or < 18 years old; (ii) were females with multiple pregnancies; (iii) had pre-existing diabetes, hypertension, acute and chronic heart, liver, kidney disease, or other serious diseases; (iv) exhibited fetal chromosomal abnormalities or major birth defects and; (v) did not have complete clinical data. According to the above criteria, 441 pregnant women with a history of GDM were included as the study group. At the same time, the mechanical sampling methods was used to sort every 40 pregnant women with no history of GDM as a list according to the medical record number. The odd-numbered pregnant women were selected. Finally, 1637 women without GDM history treated in the same period were randomly recruited as the control group. The study received ethical approval from the ethics committee of Beijing Obstetrics and Gynecology Hospital affiliated with Capital Medical University(2018-KY-030-01).

### Study design

All pregnant women with previous GDM underwent fasting plasma glucose (FPG) test at 6–8 weeks of gestation unless insulin or metformin treatment was initiated earlier. FPG ≥ 7.0 mmol/L indicates pre-gestational diabetes mellitus. These women were managed according to the standard of pre-pregnancy diabetes. The rest of pregnant women with prior GDM underwent a 75-g OGTT at 24–28 weeks of gestation, FPG ≥ 5.1 mmol/L, 1-h value ≥ 10.0 mmol/L, or 2-h value ≥ 8.5 mmol/L indicate GDM. Height and weight were recorded without shoes at the outpatient clinic or subject's home. Pre-pregnancy BMI was calculated as weight divided by height squared (kg/m^2^) using self-reported pre-pregnancy weight. Each subject in the study group received individualized counseling on diet, physical activity and weight control from trained study nurses and doctors in the first visit (usually from 8–12 weeks of gestation). A follow-up was performed every 2–4 weeks included formulating diet prescription according to pre-pregnancy body mass index (BMI) and physical activities. The control group received standard antenatal care. We used the electronic medical record system of hospital to collect patient-level variables, such as standard demographic information (age, pre-pregnancy height and weight, GWG, number of prior pregnancies, parity, family history, previous history, history of pregnancy and childbirth and education level) and laboratory data (glucose and lipid metabolism indexes at various stages of pregnancy), as well as relevant maternal and infant outcomes. Then, the above indicators of the two groups were compared statistically. Meanwhile, in women with previous GDM, we discussed the influence of GDM history on the risk of GDM and pregnancy-induced hypertension (included gestational hypertension and pre-eclampsia). We also investigated the association between GDM, pregnancy-induced hypertension and GWG before OGTT in the study group. Maternal GWG before OGTT was used to group the subjects within or above the target as recommended by the Institute of Medicine (IOM). GWG below or above the recommended threshold was defined as inadequate or excessive weight gains, respectively. The risk of GDM and pregnancy-induced hypertension in different subgroups was analyzed.

### Laboratory test indicators and pregnancy outcome

Blood lipid levels were determined at various stages of pregnancy using a full-automatic biochemical analyzer. The analysis included quantification of triglycerides (TG), total cholesterol (TC), high-density lipoprotein-cholesterol (HDL-C), and low-density lipoprotein-cholesterol (LDL-C) levels. Blood glucose levels were determined using the oxidase method during pregnancy. Gestational hypertension (GH) was defined as de novo hypertension if an individual exhibited a systolic blood pressure ≥ 140 mmHg and, or diastolic blood pressure ≥ 90 mmHg after 20 weeks of pregnancy [11], whereas pre-eclampsia (PE) was defined by the onset of hypertension and proteinuria after 20 weeks of gestation [12]. Postpartum hemorrhage (PPH) was defined as blood loss ≥ 500 mL within 24 h of delivery. Macrosomia was defined as a birth weight ≥ 4000 g, whereas preterm birth was defined as gestational age < 37 weeks. Polyhydramnios was defined as an amniotic fluid index ≥ 25 cm, whereas oligohydramnios was defined as an amniotic fluid index ≤ 5 cm. Small for gestational age (SGA) was defined as a birth weight below the 10th percentile for gestational age and gender, whereas large for gestational age (LGA) was defined as a birth weight above the 90th percentile for gestational age and gender. Lastly, premature rupture of membranes refers to a patient who is beyond 37 weeks of gestation and has presented with rupture of membranes before the onset of labor.

### Statistical analysis

All data were evaluated using the SPSS 23.0 software. Normal distribution data are presented as mean ± SD, and a t-test is utilized to compare groups. Non-normal distribution data are presented as median (25th–75th percentile) [M (P25-P75)], and the rank sum test is employed to compare between groups. Enumeration data is expressed by frequency and percentage, and the chi-square test is deployed to compare between groups. A value of p < 0.05 was regarded as statistically significant.

### Description of covariates

In 441 women with prior GDM, we performed multivariable logistic regression analysis to assess the influence of GDM history on GDM and pregnancy-induced hypertension after controlling for relevant confounding factors, such as maternal age, pre-pregnancy BMI, gravidity, parity, gestational age at delivery, cigarette smoke and alcohol consumption pre-pregnancy, family history of diabetes, glucose metabolism, lipid metabolism and GWG before OGTT. Furthermore, multivariable logistic regression analysis was applied to investigate the correlation between GDM, pregnancy-induced hypertension and GWG before OGTT of women with GDM history after controlling for relevant confounding factors, such as maternal age, pre-pregnancy BMI, gravidity, parity, gestational age at delivery, cigarette smoke pre-pregnancy and alcohol consumption pre-pregnancy.

## Results

Women with previous GDM had a significantly higher maternal age (34.8 ± 3.5 vs. 34.2 ± 3.6 years old, p = 0.002) and higher pre-pregnancy BMI (23.3 ± 3.5 vs. 22.2 ± 3.4 kg/m^2^, p < 0.001) compared with the control group. Parity (2.0 ± 0.3 vs. 2.0 ± 0.2, p < 0.001), abnormal pregnancy history (19.7% vs. 13.0%, p < 0.001), GH history (4.8% vs. 2.1%, p < 0.05), macrosomia history (7.0% vs. 1.9%, p < 0.001), and PCOS history (2.3% vs. 0.4%, p < 0.001) were significantly higher in those with prior GDM, relative to women without GDM history. Besides, a family history of type 2 diabetes was present in almost one-third of the study group participants compared to a tenth of the controls (31.3% vs. 10.8%, p < 0.001). Furthermore, women with GDM history had a higher education degree (76.2% vs. 70.7%, p < 0.05). No significant differences were seen between the two groups regarding gravidity, smoking, and PE history (p > 0.05, Table 1).

**Table 1:** Clinical characteristics, glucose and lipid indexes and pregnancy oitcomes of women with and without GDM history

	Study group	Control group	P-value
	(n = 441)	(n = 1637)	
Age, years	34.8 ± 3.5	34.2 ± 3.6	0.002
Pre-pregnancy BMI, kg/m^2^	23.3 ± 3.5	22.2 ± 3.4	< 0.001
Gravidity, %	2.9 ± 1.1	2.9 ± 1.0	0.5
Parity, %	2.0 ± 0.3	2.0 ± 0.2	0.001
Family history of diabetes, %	138 (31.3)	176 (10.8)	< 0.001
Histories of abnormal pregnancy, %	87 (19.7)	213 (13.0)	< 0.001
History of GH, %	21 (4.8)	34 (2.1)	0.002
History of PE, %	3 (0.7)	8 (0.5)	0.6
History of macrosomia, %	31 (7.0)	31 (1.9)	< 0.001
History of PCOS, %	10 (2.3)	6 (0.4)	< 0.001
Smoking, %	25 (5.7)	98 (6.0)	0.8
Education, %			
Undergraduate and above	336 (76.2)	1157 (70.7)	0.02
Junior college and below	105 (23.8)	480 (29.3)	
Glucose and lipid profiles in the first trimester, mmol/L			
TC	4.2 (3.8–4.8)	4.3 (3.8–4.8)	0.6
TG	1.3 (0.9–1.7)	1.1 (0.8–1.5)	< 0.001
HDL-C	1.5 (1.2–1.7)	1.6 (1.4–1.9)	< 0.001
LDL-C	2.2 (1.8–2.6)	2.1 (1.8–2.6)	0.1
FPG	4.9 (4.7–5.3)	4.8 (4.5–5.0)	< 0.001
Glucose and lipid profiles in the second trimester, mmol/L			
TC	6.1 (5.4–6.7)	5.8 (5.0–6.5)	< 0.001
TG	2.4 (1.9–3.0)	2.3 (1.8–2.9)	0.04
HDL-C	1.8 (1.5–1.9)	1.9 (1.7–2.2)	< 0.001
LDL-C	3.1 (2.6–3.7)	2.9 (2.4–3.5)	< 0.001
OGTT fasting glucose	4.8 (4.5–5.1)	4.5 (4.3–4.8)	< 0.001
OGTT 1-h glucose	9.1 (7.9–10.2)	7.5 (6.5–8.6)	< 0.001
OGTT 2-h glucose	7.5 (6.6–8.5)	6.4 (5.6–7.3)	< 0.001
Glucose and lipid profiles in the third trimester, mmol/L			
TC	6.4 (5.7–7.2)	6.1 (5.3–7.0)	< 0.001
TG	3.1 (2.5–3.8)	3.0 (2.4–3.7)	0.2
HDL-C	1.7 (1.5–1.9)	1.8 (1.5–2.0)	0.001
LDL-C	3.4 (2.7–4.0)	3.2 (2.5–3.8)	< 0.001
FPG	4.6 (4.3–5.0)	4.4 (4.1–4.7)	< 0.001
GWG before 16 weeks of gestation, kg	2.0 ± 2.5	2.4 ± 2.7	0.02
GWG before OGTT, kg	6.3 ± 3.3	7.9 ± 3.7	< 0.001
GWG between OGTT and delivery, kg	3.6 ± 2.1	4.0 ± 2.1	< 0.001
GWG during the entire pregnancy, kg	11.8 ± 4.6	14.4 ± 4.6	< 0.001
Gestational week at delivery, weeks	38.2 ± 1.6	38.4 ± 1.5	0.02
Delivery mode, n (%)			
Vaginal or assisted vaginal delivery	224 (50.8)	824 (50.3)	0.9
Caesarean section	217 (49.2)	813 (49.7)	
GH and PE, n (%)	37 (8.4)	88 (5.4)	0.02
GDM, n (%)	205 (46.5)	296 (18.1)	< 0.001
PPH, n (%)	67 (15.2)	293 (17.9)	0.2
Macrosomia, n (%)	46 (10.4)	148 (9.0)	0.4
Premature rupture of membrane, n (%)	84 (19.1)	275 (16.8)	0.3
Polyhydramnios and oligohydramnios, n (%)	18 (4.1)	49 (3.0)	0.3
SGA, n (%)	11 (2.5)	54 (3.3)	0.4
LGA, n (%)	57 (12.9)	158 (9.7)	0.04
Preterm birth, n (%)	34 (7.7)	94 (5.7)	0.1
Birth weight, g	3386 ± 548	3388 ± 504	0.7

In the 1st trimester of pregnancy, relative to women without GDM history, those with GDM history exhibited significantly higher FPG levels [4.9 (4.7–5.3) vs. 4.8 (4.5–5.0) mmol/L, p < 0.001] and higher TG levels [1.3 (0.9–1.7) vs. 1.1 (0.8–1.5) mmol/L, p < 0.001]. Moreover, in the 2nd trimester of pregnancy, women with previous GDM exhibited significantly higher FPG levels [4.8 (4.5–5.1) vs. 4.5 (4.3–4.8) mmol/L, p < 0.001], higher TG levels [2.4 (1.9–3.0) vs. 2.3 (1.8–2.9) mmol/L, p < 0.05], higher TC levels [6.1 (5.4–6.7) vs. 5.8 (5.0–6.5) mmol/L, p < 0.001], as well as significantly higher LDL-C levels [3.1 (2.6–3.7) vs. 2.9 (2.4–3.5) mmol/L, p < 0.001] compared with the control group. In the 3rd trimester of pregnancy, women prior GDM exhibited significantly higher FPG levels [4.6 (4.3–5.0) vs. 4.4 (4.1–4.7) mmol/L, p < 0.001], higher TC levels [6.4 (5.7–7.2) vs. 6.1 (5.3–7.0) mmol/L, p < 0.001], as well as significantly higher LDL-C levels [3.4 (2.7–4.0) vs. 3.2 (2.5–3.8) mmol/L, p < 0.001], relative to those without GDM history. Conversely, HDL-C levels throughout pregnancy were significantly lower in the study group [1.5 (1.2–1.7) vs. 1.6 (1.4–1.9) mmol/L, 1.8 (1.5–1.9) vs. 1.9 (1.7–2.2) mmol/L, 1.7 (1.5–1.9) vs. 1.8 (1.5–2.0) mmol/L, p < 0.001], relative to the control group. Nevertheless, TC and LDL-C levels in the 1st trimester of pregnancy, and TG levels in the 3rd trimester, were not significantly different (p > 0.05, Table 1).

GWG before 16 weeks of gestation, GWG before OGTT, GWG between OGTT and delivery, and GWG during entire pregnancy were significantly lower in the study group (2.0 ± 2.5 vs. 2.4 ± 2.7 kg, 6.3 ± 3.3 vs. 7.9 ± 3.7 kg, 3.6 ± 2.1 vs. 4.0 ± 2.1 kg, 11.8 ± 4.6 vs. 14.4 ± 4.6 kg, p < 0.05] relative to the control group. On the contrary, significantly higher incidence of GDM (46.5% vs. 18.1%, p < 0.001) and pregnancy-induced hypertension (8.4% vs. 5.4%, p < 0.05) were observed in the study group relative to the control group. The multivariate logistic regression results demonstrated that women with prior GDM had a higher risk of developing GDM (OR 3.25, 95% CI 2.26–4.68) and pregnancy-induced hypertension (OR 1.50, 95% CI 1.05–2.45) compared with those without previous GDM. Besides, relative to women without previous GDM, the LGA rate was higher in those with GDM history (12.9% vs. 9.7%, p < 0.05). On the contrary, the gestational week at delivery was lower in those with GDM history (38.2 ± 1.6 vs. 38.4 ± 1.5 weeks, p < 0.05). Delivery mode, PPH, macrosomia, premature membrane rupture, polyhydramnios and oligohydramnios, SGA, preterm birth, and birth weight were not significantly different (p > 0.05, Table 1, 2).

**Table 2:** The relationship between pregnancy outcomes and GDM history

	Without GDM history	With GDM history
		Adjusted OR 95% CI
	(N = 1637)	(N = 441)
GDM	1	3.25 [2.26–4.68]**
GH and PE	1	1.50 [1.05–2.45]*
LGA	1	1.34 [0.96–1.87]

Data was analyzed using multivariable logistic regression analysis. Models were adjusted for maternal age, pre-pregnancy BMI, gravidity, parity, gestational age at delivery, cigarette smoke pre-pregnancy, alcohol consumption pre-pregnancy, family history of diabetes, glucose and lipid metabolism and GWG in the 1st and 2nd trimesters

Reference group: women without GDM history

*OR* odds ratio, *CI* confidence interval

* p < 0.05

** p < 0.005

Table 3 shows the relationship between GDM, pregnancy-induced hypertension and GWG before OGTT in women with GDM history. Multivariate logistic regression demonstrated that compared with women in the inadequate GWG group, individuals in the excessive GWG group had a higher risk of developing pregnancy-induced hypertension (OR 1.47, 95% CI 1.05–3.32), while inadequate GWG was not a protective factor for GDM and pregnancy-induced hypertension (Table 3).

**Table 3:** The relationship between GDM, pregnancy-induced hypertension and GWG before OGTT in women with GDM history

GWG before OGTT	Inadequate (N = 122)	Excessive (N = 119)
	Adjusted OR 95% CI	Adjusted OR 95% CI
GDM	0.93 [0.56–1.55]	1.08 [0.65–1.81]
GH and PE	0.34 [0.11–1.11]	1.47 [1.05–3.32]*

Data was analyzed using multivariable logistic regression analysis. Models were adjusted for maternal age, pre-pregnancy BMI, gravidity, parity, gestational age at delivery, cigarette smoke pre-pregnancy and alcohol consumption pre-pregnancy

Reference group: adequate GWG group in women with GDM history

*OR* odds ratio, *CI* confidence interval

* p < 0.05

** p < 0.005

## Discussion

Our data suggested that FPG during the entire pregnancy, TG levels in the 1st and 2nd trimesters, TC and LDL-C levels in the 2nd and 3rd trimesters in women with previous GDM were higher than those without GDM history. Inversely, relative to the control group, women with GDM history exhibited significantly lower HDL-C levels during the whole pregnancy. Intriguingly, although GWG at various pregnancy stages were lower in women with previous GDM than those without GDM history, we still found a higher risk of GDM and pregnancy-induced hypertension in women with prior GDM. Furthermore, in women with GDM history, excessive GWG increased the risk of developing pregnancy-induced hypertension, while insufficient GWG had no protective effect on the occurrence of GDM and pregnancy-induced hypertension.

The high risk of type 2 diabetes and recurrence in subsequent pregnancies has been well documented in women with GDM history [13, 14], and the recurrence rate may vary according to ethnic differences and characteristics of participants (30–84%) [15, 16]. Prior studies found a high risk of type 2 diabetes and abnormal glucose tolerance in women with previous GDM, with an incidence of up to four times greater than general women [17]. Consistent with previous studies, our data indicated that women with previous GDM had a threefold risk for GDM (OR = 3.25, 95% CI 2.26–4.68) relative to counterparts without GDM history. Our study also demonstrated that GDM history is one of the risk factors of GH and PE. In particular, the risk of developing GH and PE in women with prior GDM is 1.50 (OR = 1.50, 95%CI 1.05–2.45) times higher than the control group. Mounting evidence suggested that women with GDM history are at higher risk of metabolic syndrome and cardiovascular disease, including hypertension, dyslipidemia, and obesity [18–20]. Hromadnikova et al. [21] uncovered long-term epigenetic changes in mothers' peripheral blood with GDM history that may predispose them to diabetes, cardiovascular, and cerebrovascular diseases. The dyslipidemia characteristics in our paper may be one reason for the high incidence of pregnancy-induced hypertension in women with a history of GDM.

Persistent high insulin resistance during pregnancy can stress and exacerbate pancreatic cells’ damage [22, 23]. Multiple studies revealed that women with GDM had varying insulin resistance levels and reduced insulin sensitivity after delivery [24–27]. A Greek study found that women with previous GDM presented various degrees of impaired fasting glucose and impaired glucose tolerance in postpartum [28]. Herein, we did not measure insulin levels; however, our data illustrated that women with previous GDM exhibited significantly higher FPG in early pregnancy stages than the control group. It was suggested that abnormal glucose metabolism in GDM may not end with delivery but persist postpartum and carry the condition into subsequent pregnancies. Meanwhile, our study also indicated that abnormal lipid metabolism exists at the early stage of pregnancy in the study group, including higher TG, TC, LDL-C, and lower HDL-C levels. Similarly, numerous studies also confirmed that dyslipidemia increases the risk of GDM [29, 30]. This may be attributed to the following two reasons. Excess TG was stored in skeletal muscles, which may impair insulin signaling, and eventually, the lipotoxic effect of FFAs leads to decreased insulin-stimulated skeletal muscle glucose transport [31]. In addition, oxidative stress, secondary to dyslipidemia, may ultimately cause decreased insulin gene expression and insulin secretion impairment [32].

Excessive GWG is a well-established risk factor for GDM [33–36]. We evaluated GWG at various pregnancy stages in two groups of women. Our findings suggested that GWG before 16 weeks and GWG before OGTT were significantly lower in women with GDM history than those without GDM history. Consistent with our results, Persson et al. [37] also found that women with GDM history gained less GWG than those without GDM history, related to the extensive intervention of GWG initiated from early pregnancy. However, they did not analyze GWG in various pregnancy stages, which is discussed in our article for the first time. In our hospital, women with previous GDM were provided individualized medical nutritional therapy (MNT) and exercise guidance to help control GWG in the first visit (usually from 8–12 weeks of gestation). To prevent GDM recurrence in these women, a follow-up was performed every 2–4 weeks, including formulating diet prescription according to BMI and physical activities. Once GDM was diagnosed, in addition to MNT, more stringent weight gain targets were set, and insulin therapy was given when necessary. From our data, our intervention resulted in significantly lower GWG before 16th weeks of gestation (0.4 kg less than the control group in this study) and before OGTT (1.6 kg less than the control group in this study) in women with previous GDM than those without GDM history. Nevertheless, in the present study, GDM incidence in women with GDM history is still much higher than in the control group. Comparably and of particular note, our findings further indicated that excessive GWG before OGTT exhibited a significantly positive correlation with pregnancy-induced hypertension in women with previous GDM, compared with GWG within the IOM guidelines in women with GDM history. Nevertheless, inadequate GWG before OGTT did not have a protective effect on GDM and pregnancy-induced hypertension. Our finding was inconsistent with previous studies involving general pregnant women [38]. Whether the complicated endocrine and metabolism in women with GDM history are involved should be further investigated. Besides, this seems to suggest that a limited reduction of GWG before OGTT may not be enough to offset the effects of pre-existing impaired glucose and lipid metabolism on adverse pregnancy outcomes in women with GDM history. However, the superposition effect of GDM history and excessive GWG state may contribute to increasing pregnancy-induced hypertension. Similar to our findings, a multicenter randomized controlled trial in Europe indicated that lifestyle change, even with an average 4.3 kg GWG reduction in the combined diet and exercise interventions by 35–37 weeks, is insufficient to prevent GDM in overweight/obese women [39]. Whether a larger amount of GWG reduction before OGTT or initiation of intervention before conception may result in a decline in GDM in women with GDM history remains unknown, which urgently needs to be explored in the future.

### Strength and limitation

The strength of this study lies in having a larger sample size, which fully integrated data on glucose and lipid metabolism, and GWG at various pregnancy stages for both groups. Furthermore, for the first time, we conducted a subgroup analysis of GWG extent in women with GDM history and found that even a 1.6 kg reduction in GWG before OGTT did not reduce the risk of GDM and pregnancy-induced hypertension. However, our study has limitations. This study was not a randomized controlled trial but a retrospective analysis. Moreover, the study was limited to single-center data, indicating that our findings may not reflect individuals from other Chinese regions or countries, owing to potential differences in education and socioeconomic levels. The current data were also insufficient to answer definitively essential questions of how much reduction of GWG or when to intervene can improve the adverse outcomes.

## Conclusion

In summary, for women with GDM history, the superposition effect of advanced age, higher pre-pregnancy BMI, as well as glucose and lipid metabolism disorders in the 1st trimester can lead to adverse pregnancy outcomes. Limited reduction of GWG may not be enough to reduce the incidence of adverse pregnancy outcomes in those with GDM history. Relevant and necessary management may need to be initiated at early stage or even before pregnancy. Furthermore, it is unclear how much GWG during pregnancy and what kind of patterns of GWG are applicable to women with GDM history. Therefore, large sample-sized and multicenter studies need to confirm when and what kind of intervention intensity is appropriate for women with previous GDM.

## Data Availability

The data is available upon reasonable request to the corresponding author.

## Abbreviations

GDM: Gestational diabetes mellitus
GWG: Gestational weight gain
OGTT: Oral glucose tolerance test
FPG: Fasting plasma glucose
BMI: Body mass index
IOM: Institute of Medicine
TG: Triglycerides
TC: Total cholesterol
HDL-C: High density lipoprotein-cholesterol
LDL-C: Low density lipoprotein-cholesterol
GH: Gestational hypertension
PE: Preeclampsia
PPH: Postpartum hemorrhage
SGA: Small for gestational age
LGA: Large for gestational age
OR: Odds ratio
CI: Confidence interval
